# Association Between Behavioral, Biological, and Genetic Markers of Cardiovascular Health and MRI Markers of Brain Aging: A Cohort Study

**DOI:** 10.1212/WNL.0000000000201346

**Published:** 2023-01-03

**Authors:** Yuanjing Li, Erika J. Laukka, Serhiy Dekhtyar, Goran Papenberg, Andreja Speh, Laura Fratiglioni, Grégoria Kalpouzos, Chengxuan Qiu

**Affiliations:** From the Aging Research Center (Y.L., E.J.L., S.D., G.P., A.S., L.F., G.K., C.Q.), Department of Neurobiology, Care Sciences and Society, Karolinska Institutet-Stockholm University, Stockholm, Sweden; Stockholm Gerontology Research Center (E.J.L., L.F.), Stockholm, Sweden; Department of Neurology (A.S.), University Medical Center Ljubljana, Ljubljana, Slovenia; and Medical Faculty (A.S.), University of Ljubljana, Ljubljana, Slovenia.

## Abstract

**Background and Objective:**

The life’s simple 7 approach was proposed to define cardiovascular health (CVH) metrics. We sought to investigate the associations between behavioral, biological, and genetic markers for CVH and vascular brain aging in older adults.

**Methods:**

This population-based cohort study included participants who had repeated brain MRI measures from 2001 to 2003 to 2007–2010 (i.e., count of perivascular spaces, volumes of white matter hyperintensity [WMH] and gray matter, and lacunes). At baseline, global, behavioral, and biological CVH metrics were defined and scored following the life’s simple 7 approach and categorized into unfavorable, intermediate, and favorable profiles according to tertiles. The metabolic genetic risk score was calculated by counting 15 risk alleles associated with hypertension, diabetes, or dyslipidemia. Data were analyzed using linear mixed-effects and Cox proportional hazards models, adjusting for age, sex, and education.

**Results:**

The study sample consisted of 317 participants (age 60 years or older; 61.8% women). Favorable and intermediate (vs unfavorable) global CVH profiles were related to slower WMH progression, with β-coefficients (95% CI) being −0.019(-0.035–0.002) and −0.018(-0.034–0.001), respectively. Favorable and intermediate (vs unfavorable) biological CVH profiles were significantly related to slower WMH increase only in people aged 60–72 years. CVH profiles were not related to progression of other brain measures. Furthermore, a higher metabolic genetic risk score (range: 6–21) was associated with faster WMH increase (β-coefficient = 0.005; 95% CI: 0.003–0.008). There were statistical interactions of metabolic genetic risk score with global and behavioral CVH profiles on WMH accumulation. A higher metabolic genetic risk score was related to faster WMH accumulation, with β-coefficients being 0.015(0.007–0.023), 0.005(0.001–0.009), and 0.003(-0.001 to 0.006) among people with unfavorable, intermediate, and favorable global CVH profiles, respectively; the corresponding β-coefficients were 0.013(0.006–0.020), 0.006(0.003–0.009), and 0.002(-0.002 to 0.006) among people with unfavorable, intermediate, and favorable behavioral CVH profiles.

**Discussion:**

Intermediate to favorable global CVH profiles in older adults are associated with slower vascular brain aging. The association of metabolic genetic risk load with accelerated vascular brain aging was evident among people with unfavorable to intermediate, but not favorable, CVH profiles. These findings highlight the importance of adhering to favorable CVH profiles, especially healthy behaviors, in vascular brain health.

In 2010, the American Heart Association proposed the life’s simple 7 approach to defining cardiovascular health (CVH) based on 4 behavioral factors (i.e., smoking, physical activity, diet habit, and body mass index) and 3 biological factors (i.e., total cholesterol, blood pressure, and fasting plasma glucose).^1^ Several cohort studies found that ideal or favorable CVH metrics were associated with slower cognitive decline and a reduced risk of dementia in late life,^2-7^ suggesting that favorable CVH metrics may benefit brain health in old age. Indeed, cross-sectional data from the Northern Manhattan Study suggested that the favorable levels of CVH metrics were associated with lower volume of white matter hyperintensity (WMH), higher volume of total brain tissue, and a reduced likelihood of silent brain infarcts in older people.^8^ Yet, longitudinal data are scarce with respect to the associations of CVH metrics with structural measures of brain aging, such as WMH, perivascular space (PVS), lacune, and brain volume.^9^ Furthermore, population-based studies have shown evidence that favorable CVH metrics in midlife or earlier older adulthood, but not in later older adulthood, are associated with slower cognitive decline and a reduced risk of dementia.^3,7^ However, it is unclear whether the associations of CVH metrics with brain aging vary with age.

Previous studies have suggested that metabolic factor-related susceptibility genes, such as *APOE* (a susceptibility gene to dyslipidemia) and *ACE* (a susceptibility gene to hypertension), are associated with loads of cerebral WMH and microbleeds.^10,11^ However, whether a cluster or concurrent presence of several metabolic susceptibility genes may have cumulative effects on vascular brain aging remains to be elucidated. Furthermore, given that adapting healthy behaviors (e.g., physical activity) might partly offset the detrimental effect of genetic susceptibility (e.g., *APOE* ε4) on brain aging and cognitive outcomes,^12,13^ it is plausible to hypothesize that the favorable level of CVH metrics may modify the association of metabolic susceptibility genes with vascular brain aging. However, evidence supporting this hypothesis remains lacking.

In this population-based cohort study of Swedish older adults, we aimed to investigate the associations of CVH metrics and metabolic susceptibility genes with vascular brain aging. We hypothesized that 1) the favorable level of CVH metrics would be associated with a lower rate of vascular brain aging, in which the association may vary by age; 2) higher genetic susceptibility to metabolic risk factors would be associated with faster progression of vascular brain aging; and 3) the association of higher metabolic genetic predisposition with vascular brain aging might be partly mitigated among people with favorable level of CVH metrics.

## Methods

### Study Design and Participants

This is a population-based cohort study. The study participants were derived from the magnetic resonance imaging (MRI) substudy of the population-based Swedish National Study on Aging and Care in Kungsholmen (SNAC-K).^14^ In brief, SNAC-K is a multidisciplinary study of aging and health among people 60 years and older in the Kungsholmen district of Stockholm, Sweden. In 2001–2004, 3363 noninstitutionalized residents 60, 66, 72, 78, 81, 84, 87, 90, 93, 96, and 99 years or older, underwent the baseline examinations. Of these, 555 nondisabled participants underwent brain structural MRI examinations in 2001–2003.^14,15^

**Figure 1:**
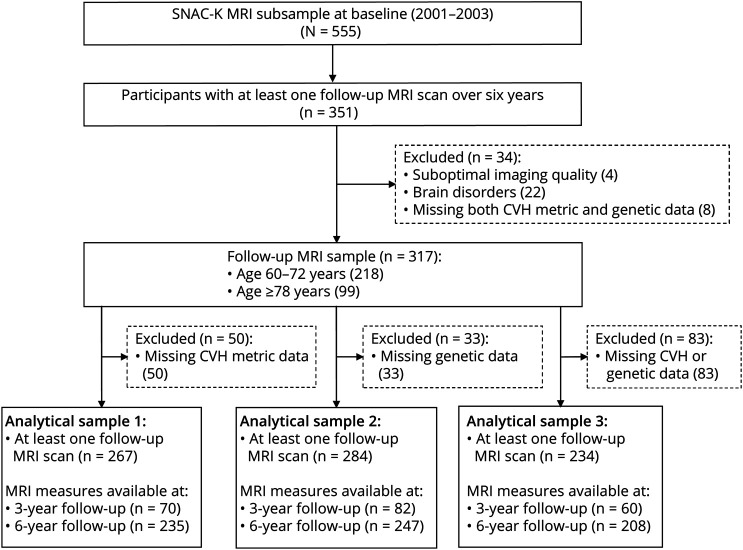
Flowchart of Study Participants in SMAC-K MRI Substudy, 2001–2003 and 2007-2010 SNAC-K = Swedish National Study on Aging and Care in Kungsholmen; MRI = magnetic resonance imaging; CVH = cardiovascular health.

The follow-up MRI examinations were performed in 2007–2010 for participants aged 60–72 years and in 2004–2007 and 2007–2010 for those 78 years or older, according to the overall follow-up scheme of SNAC-K.^14^ Of the 555 MRI participants at baseline, 351 had at least 1 follow-up MRI scan over 6 years; among them, 34 were excluded due to incomplete or suboptimal quality of images (n = 4); brain infarcts, brain tumors, or arachnoid cysts (n = 21); probable dementia (n = 1); or missing both CVH profile scores and genetic risk scores (n = 8), leaving 317 persons for the current analyses; among them, data were available in 267 people for composite CVH profiles (analytical sample 1), in 284 people for composite genetic scores (analytical sample 2), and in 234 people for both composite CVH profiles and genetic scores (analytical sample 3). Figure 1 shows the flowchart of the study participants.

### Standard Protocol Approvals, Registrations, and Patient Consents

All phases of data collection in SNAC-K and the linkages of SNAC-K data with patient register and death register were approved by the Ethics Committee at Karolinska Institutet or the Regional Ethical Review Board in Stockholm. Written informed consents were obtained from all participants before data collection.

### Data Collection and Assessment of Composite CVH Metrics at Baseline

At baseline, data on demographics (age, sex, and education), lifestyle (e.g., smoking and physical activity), cardiometabolic risk factors (e.g., hypertension, dyslipidemia, and diabetes), health conditions, and use of medications were collected through face-to-face interviews, neuropsychological testing, clinical examinations, laboratory tests, and the linkage with the Swedish National Patient Register and death register.^14^ All medications were classified and coded according to the Anatomical Therapeutic Chemical (ATC) Classification System.

The operational approaches for defining and categorizing CVH metrics in SNAC-K were previously described in detail.^7^ In brief, we defined and categorized each of the 7 individual CVH metrics as poor (score = 0), intermediate (score = 1), and ideal (score = 2) levels, respectively, following the life’s simple 7 approach proposed by the American Heart Association,^1^ with modifications on smoking status, diet, and blood glucose levels,^7^ according to data available in our project. We categorized smoking status into current smoking, stopped smoking in the past 5 years, and stopped 5 years ago or never smoking. We categorized physical activity into physical inactivity (never or ≤3 times per month), light exercise (e.g., walks, short bike rides, light aerobic activities or gym classes, and golf at least once a week), and moderate-to-intense exercise (e.g., brisk walking, jogging, heavy gardening, long bike rides, intense aerobic activities or gym classes, skating, skiing, swimming, and ball games or similar activities at least once a week). We assessed diet habits according to the 98-item semiquantitative food frequency questionnaire, which included the intake frequencies of each food item over the past year.^16^ Body mass index was calculated as measured weight (kg) divided by height (m) squared. Arterial blood pressure was measured twice on the left arm in a sitting position using the sphygmomanometer, and the mean of 2 readings was used for the analysis. Nonfasting total serum cholesterol was initially measured, and if the nonfasting total cholesterol was higher than 6.5 mmol/L, then the fasting total cholesterol was measured, and the mean of both measurements was used in the analysis. Glycated hemoglobin A1c (HbA1c) was measured using the Swedish Mono-S High Performance Liquid Chromatography and added by 1.1% to conform to international values, according to the National Glycohemoglobin Standardization Program.^17^ Diabetes was diagnosed as HbA1c ≥ 6.5%, current use of hypoglycemic medication (ATC code: A10) or having records of diabetes in the National Patient Register. Prediabetes was defined as having the level of HbA1c between 5.7%–6.5% among participants without diabetes.^7^ eTable 1, links.lww.com/WNL/C361, presents detailed descriptions on the definitions and scores of 7 CVH metrics.

We estimated the behavioral metric score (score range in the SNAC-K total sample: 0–8) by adding up scores of 4 health behaviors (smoking status, physical activity, diet, and body mass index) and the biological metric score (score range in the SNAC-K total sample: 0–6) by adding up scores of 3 biological health factors (blood pressure, total cholesterol, and blood glucose). The global CVH metric score (score range in the SNAC-K total sample: 0–14) was estimated by adding up scores of both the behavioral and biological CVH metrics. Then, according to tertiles of respective composite CVH metric score among baseline participants in the SNAC-K total sample, we categorized them into unfavorable (lower tertile), intermediate (medium tertile), and favorable (upper tertile) profiles.^7^

### Assessment of Metabolic Genetic Risk Score

DNA was extracted from whole blood samples. Genotyping was performed using MALDI-TOF analysis on the Sequenom MassARRAY platform at the Mutation Analysis Facility, Karolinska Institutet.^18^ In total, 103 single nucleotide polymorphisms (SNPs) that were potentially associated with cognitive phenotypes in aging, cardiovascular risk, and longevity were genotyped in SNAC-K. We selected 15 SNPs from these SNPs for estimating the metabolic genetic risk score. The selected SNPs were related to hypertension, dyslipidemia, and diabetes, which corresponded to the 3 biological health metrics in the life’s simple 7 approach, based on the findings from previous genome-wide association studies, fine-mapping analyses, or meta-analyses, otherwise from at least 2 different original studies (i.e., *LIPC* rs1800588 and *LDLR* rs5930 as dyslipidemia susceptibility genes) (eTable 2, links.lww.com/WNL/C361). The distributions of all these genotypes conformed to the Hardy-Weinberg equilibrium (*p* > 0.05). The metabolic genetic risk score was created by adding up the number of risk alleles of single nucleotide polymorphisms related to hypertension, dyslipidemia, or diabetes.^19^ *APOE* genotype, determined by rs429358 and rs7412, was scored by counting the number of ε4 alleles (score range: 0–2). We categorized the metabolic genetic risk score into low, intermediate, and high metabolic genetic risk loads according to tertiles of the score.

### Acquisition and Evaluation of MRI Measures

All eligible participants underwent brain MRI scans on a 1.5T system (Philips Intera, The Netherlands). The same scanner and the same parameters were used at baseline and all follow-up MRI examinations.^15^ The core sequences included a MPRAGE T1-weighted sequence (resolution: 0.94 × 0.94 × 1.5 mm; no gap; repetition time, 15 ms; echo time, 7 ms; flip angle, 15°), a proton density/T2-weighted sequence (resolution: 0.98 × 0.98 × 3 mm; no gap; repetition time, 3995 ms; echo time, 18/90 ms; echo train length, 6; flip angle, 90°), and a fluid-attenuated inversion recovery (FLAIR) sequence (resolution: 0.90 × 0.90 × 5 mm; gap: 1 mm; repetition time, 6,000 ms; echo time, 100 ms; echo train length, 21; flip angle 90°).

A trained rater (Y.L.) visually evaluated PVS and lacunes under the supervision of a senior neuroimaging analyst (G.K.), as previously reported.^15^ In brief, PVS refers to the fluid-filled cavity surrounding the arterials, venules, and capillaries because they penetrate from the subarachnoid space through brain parenchyma. PVS is visualized as the cerebrospinal fluid intensity with a diameter within 3 mm when imaged perpendicularly or linear when imaged in parallel to the course of perforating vessels.^9^ The number of PVS was counted in the frontal lobe, parieto-occipital lobe, cerebellum, mesencephalon, and hippocampus using the axial T2-weighted sequence and counted in the basal ganglia and subinsular region using the axial T1-weighted sequence because here T1 image presented a stronger contrast between intensities of PVS and background tissues, compared with T2 image. For each region, PVS was recorded in the slice with the highest numbers, following a validated protocol.^20^ The global PVS count was the sum of all above regional PVS counts on both hemispheres. Lacune of vascular origin presents as a round or ovoid fluid-filled cavity, 3–15 mm in diameter in the territory of perforating arterioles.^9^ Both number and location of lacune were recorded using FLAIR and axial T2-weighted images. We defined prevalent lacune as any lacune identified at baseline. We considered any newly emerged lacune detected on the follow-up images as an incident lacune. One month after the initial evaluation, reassessments of 30 randomly selected brain images by the rater (Y.L.) yielded a correlation coefficient of 0.91 for global PVS count and a weighted κ of 0.81 for lacunes (intrarater reliability).^15^

The senior neuroimaging analyst (G.K.) manually drew WMH on FLAIR images and further interpolated them on the corresponding T1-weighted images to compensate for the gap between slices in FLAIR, using MRIcron (nitrc.org/projects/mricron).^21^ Then, the global WMH volume was automatically estimated in MRIcron and was log-transformed due to its right-skewed distribution. Gray matter (GM) volume and total intracranial volume on T1-weighted images were automatically assessed in Statistical Parametric Mapping (SPM - Statistical Parametric Mapping (ucl.ac.uk)).^14^ GM volume was adjusted by total intracranial volume using linear regression.^22^ We did not adjust WMH volume using total intracranial volume because the log-transformed WMH volume was not associated with total intracranial volume.

### Statistical Analyses

Baseline characteristics of study participants by unfavorable, intermediate, and favorable global CVH profiles were compared using general linear models for continuous variables and the χ^2^ test for categorical variables. In analytical sample 1, we assessed the associations of CVH profiles with annual changes (β-coefficients related to CVH profiles × follow-up time [years]) in continuous brain measures (i.e., WMH volume, PVS count, and GM volume) using linear mixed-effects models. The linearity in the changes of these continuous brain variables during the follow-up period was verified in the previous report.^15^ We assessed the associations of CVH profiles with incident lacunes using Cox proportional hazards models. Then, the 3-item interaction of CVH profiles, age groups (60–72 vs 78 years or older), and follow-up time on brain measures was tested using linear mixed-effects models. Similarly, in analytical sample 2, we examined the associations of the metabolic genetic risk score with annual progression (β-coefficients of metabolic genetic risk score × years of follow-up time) of continuous brain measures using linear mixed-effects models. We assessed the associations of the metabolic genetic risk score with incident lacunes using Cox proportional hazards models. Next, in the analytical sample 3, we examined the 3-item interaction of the metabolic genetic risk score, CVH profiles, and follow-up time on changes of continuous brain measures, using linear mixed-effects models. When a statistical interaction was detected (*p* for interaction<0.05), we further performed the stratified analyses by CVH profiles to assess the direction and magnitude of the association between metabolic genetic risk score and structural brain measures. In the sensitivity analysis, given the evident effect of *APOE* gene on brain aging,^11^ we repeated the aforementioned analyses by excluding *APOE* gene from the metabolic genetic risk score. In addition, to assess the impact of the genetic susceptibility to brain aging per se on the results, we further adjusted for a composite genetic risk score for brain aging, which was generated from 15 SNPs available in our data set that were potentially related to MRI markers of brain aging (e.g., WMH, PVS, and brain atrophy). Stata Statistical Software: Release 16.0 for Windows (StataCorp LLC, College Station, TX, USA) was used for all the analyses.

### Data Availability

Data on which this study is based are derived from the population-based SNAC-K project (snac-k.se/). Access to these anonymized SNAC-K data will be available on reasonable request and approval by the SNAC-K data management and maintenance committee at the Aging Research Center, Karolinska Institutet, Stockholm, Sweden.

## Results

### Baseline Characteristics of Study Participants

Of the 3363 participants in SNAC-K, 555 undertook the brain MRI scans at baseline. Compared with people who did not have MRI scans (n = 2,808), those who undertook MRI scans were younger (mean age: 71.2 vs 75.4 years, *p* < 0.001), more educated (university degree: 41.0% vs 30.7%, *p* < 0.001), and more likely to be male (41.8% vs 33.8%, *p* = 0.001). At baseline, of the 267 participants in the analytical sample 1, the global CVH metric score ranged from 2 to 13, with the mean score being 7.91 (standard deviation [SD] = 1.89). The mean global CVH metric scores (SD) in participants with unfavorable, intermediate, and favorable global CVH profiles were 5.31 (0.86), 7.50 (0.50), and 9.78 (0.95), respectively. There were no significant differences in mean age and distribution of sex and education by global CVH profiles (Table 1). In the analytical sample 2, the metabolic genetic risk score was available in 284 participants, with the score ranging from 6 to 21 (mean score = 13.03; SD = 2.39).

**Table 1:**
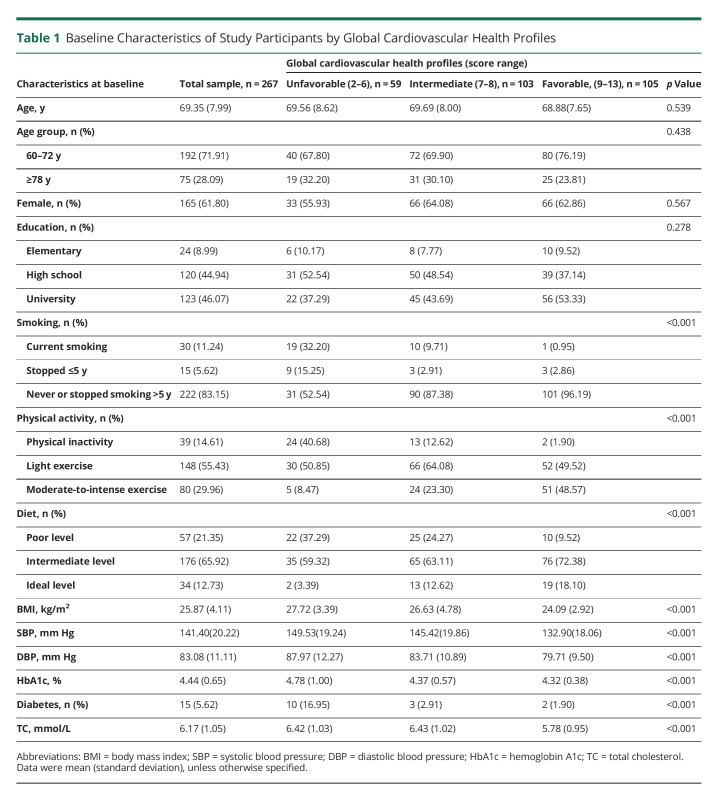
Baseline Characteristics of Study Participants by Global Cardiovascular Health Profiles

### Associations of CVH Profiles With Markers of Vascular Brain Aging (Analytical Sample 1, N = 267)

The average follow-up time was 5.47 years (SD = 0.90). Higher global and behavioral CVH metric scores were related to a faster increase of global WMH volume (*p* < 0.01, Table 2). When categorizing CVH metric score into tertiles, the intermediate and favorable (vs unfavorable) global CVH profiles were significantly related to less annual increase in global WMH volume (*p* < 0.05); the intermediate, but not favorable, behavioral CVH profile was significantly related to slower progression of WMH volume (*p* < 0.001, Table 2). Global and behavioral CVH profiles had no significant associations with annual changes of PVS count, GM volume, or incidence of lacunes (Table 2). For the biological CVH profiles, the intermediate and favorable (vs unfavorable) profiles were significantly related to slower GM atrophy (Table 2).

**Table 2:**
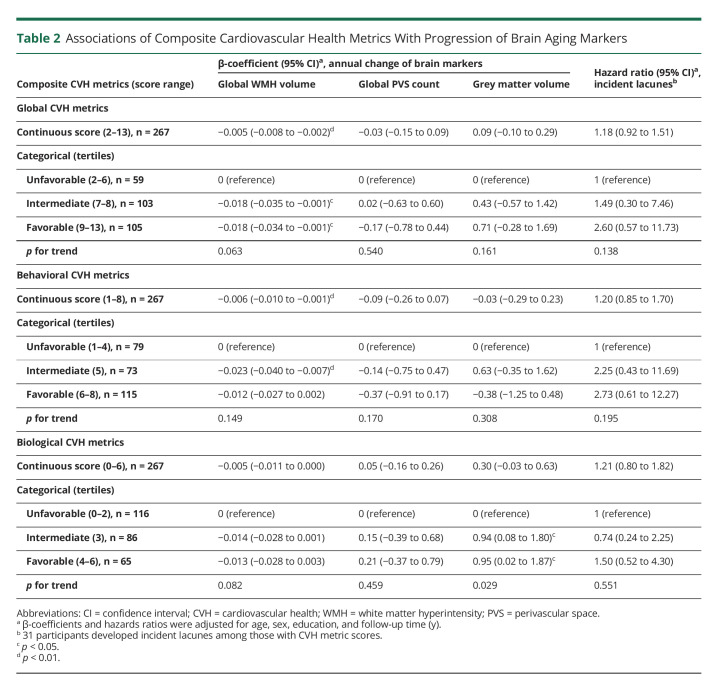
Associations of Composite Cardiovascular Health Metrics With Progression of Brain Aging Markers

**Figure 2:**
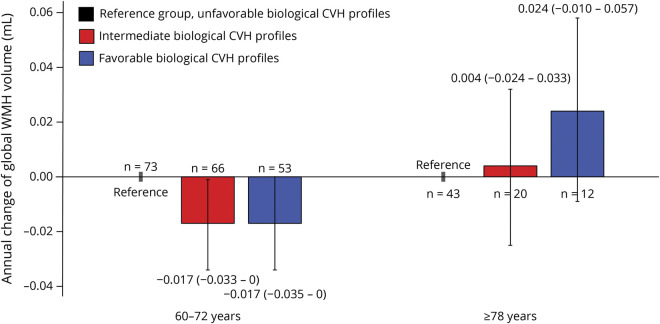
Associations of Biological CVH Profiles With Annual Changes of Global WMH Volume by Age Groups (Analytical Sample 1) CVH = cardiovascular health; WMH = white matter hyperintensity.

We detected a marginally statistical interaction of biological CVH metric score, age groups (60–72 vs 78 years or older), and follow-up time on changes of WMH volume (*p* for interaction = 0.062). Stratified analysis by age groups suggested that each 1-point increment in biological CVH metric score was significantly associated with slower accumulation of WMH among people aged 60–72 years (β-coefficient = −0.007; 95% confidence interval −0.013 to 0.000; *p* = 0.042) but not among those 78 years or older (0.008; −0.004 to 0.019). As a categorical variable, intermediate and favorable (vs unfavorable) biological CVH profiles were significantly associated with slower accumulation of WMH over time among people aged 60–72 years but not among those 78 years or older (Figure 2).

### Associations of Metabolic Genetic Risk Loads With Markers of Vascular Brain Aging (Analytical Sample 2, N = 284)

A higher metabolic genetic risk score was significantly associated with a faster accumulation of WMH over time (*p* < 0.001, Table 3). There were no significant associations between metabolic genetic risk score and annual progression of PVS count, GM volume, and lacunes (Table 3). When categorizing the metabolic genetic risk score into tertiles, the high genetic risk load (vs low) was related to a faster progression of WMH (*p* for linear trend = 0.002, Table 3). The intermediate genetic risk load (vs low) was also related to faster GM atrophy (*p* < 0.05, Table 3). The associations between metabolic genetic risk loads and volumes of WMH and GM remained significant after removing *APOE* gene from the metabolic genetic risk score (data not shown). Furthermore, additional adjustment for a composite genetic risk score for brain aging did not substantially affect the observed associations (data not shown).

### Association of Metabolic Genetic Risk Load With WMH Progression by Levels of CVH Profiles (Analytical Sample 3, N = 234)

**Figure 3:**
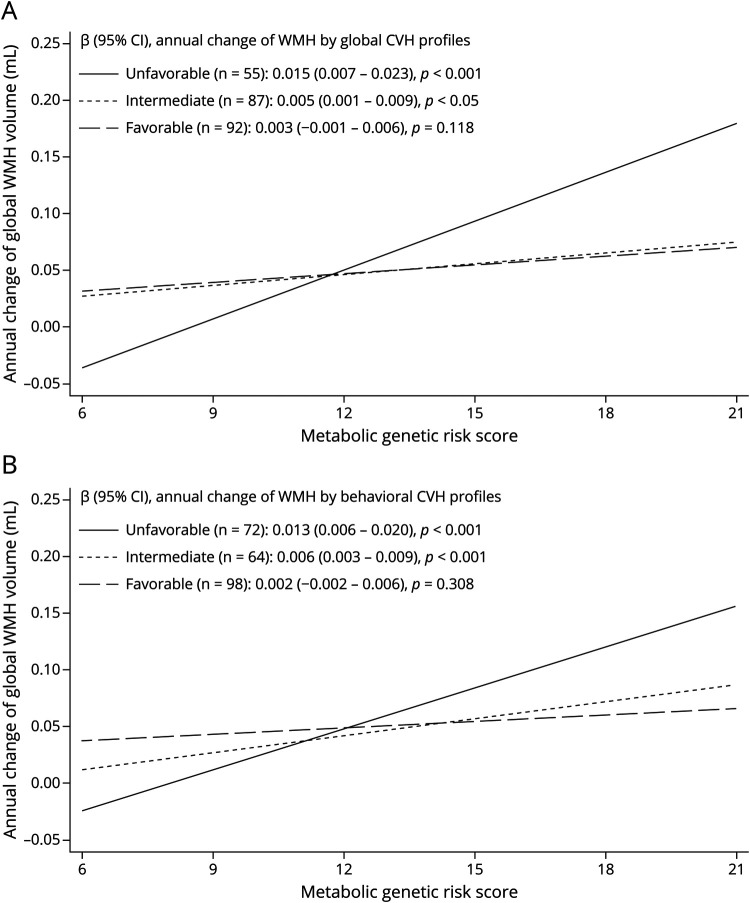
Associations of Genetic Susceptibility to Metabolic Risk Factors With Annual Changes of Global WMH Volume by CVH Profile Categories: β-coefficients and 95% Confidence Intervals Were Adjusted for Age, Sex, and Education (Analytical Sample 3) (A) Association of metabolic genetic risk score with annual changes of global WMH volume by global CVH profiles (n = 234). (B) Association of metabolic genetic risk score with annual changes of global WMH volume by behavioral CVH profiles (n = 234).

We detected statistical interactions of global and behavioral CVH metric scores with metabolic genetic risk score on annual progression of WMH (*p* for both interactions = 0.001). Higher metabolic genetic risk score was associated with faster progression of WMH in people with unfavorable and intermediate global CVH profiles, but not in those with favorable global CVH profiles (Figure 3A). Similarly, an increased metabolic genetic risk load was significantly related to a faster increase of WMH in people with unfavorable and intermediate behavioral CVH profiles, but not in those with favorable behavioral CVH profiles (Figure 3B). The statistical interactions of CVH profiles with the metabolic genetic risk score remained significant even after removing *APOE* gene from the genetic risk score or further adjusting for a composite genetic risk score for brain aging (data not shown).

## Discussion

In this population-based cohort study of Swedish older adults, we investigated the associations of CVH profiles with genetic susceptibility to metabolic risk factors with markers of vascular brain aging. We found that (1) intermediate to favorable (vs unfavorable) global CVH profiles were associated with slower progression of global WMH; (2) intermediate to favorable biological CVH profiles were related to slower progression of global WMH among people aged 60–72 years, but not among those 78 years or older; and (3) a higher genetic predisposition to metabolic risk factors was associated with faster accumulation of global WMH, whereas such association was not evident among people with favorable global and behavioral CVH profiles.

Our findings highlight the potential role of favorable CVH profiles in maintaining vascular brain health in older adults. However, the Offspring Cohort of Framingham Heart Study in middle-aged people (45 years or older) did not find the association between ideal composite CVH metrics and slower WMH accumulation,^4^ which differs from our study of older adults. People in middle age usually have milder WMH burden compared with older adults, which might partly contribute to the discrepancies. In addition, the Framingham Offspring Study found that ideal composite CVH metrics were related to slower brain atrophy over 10 years, suggesting that a long-term follow-up period may be critical to see an association with brain atrophy. Data from the population-based cross-sectional study in the United Kingdom showed that healthy behavior factors (e.g., normal body mass index and regular physical activity) were associated with low burden of WMH,^23^ supporting the potential role of favorable behavioral CVH profile in maintaining vascular brain health. This is in line with our findings. Hypertension and diabetes are known to be associated with accelerated WMH accumulation.^24,25^ These cardiometabolic risk factors could remodel structure of cerebral blood vessels, thus limiting blood flow, facilitating arteriosclerosis, and reducing oxygen and glucose to the brain.^26,27^ Therefore, it is biologically plausible that favorable biological CVH profiles are associated with vascular brain health. We did not find any association of CVH profiles with annual PVS changes. Similarly, previous population-based studies indicated that cardiovascular risk factors were not related to PVS, suggesting that the potential nonvascular pathogenic mechanisms might underlie the development of PVS.^28,29^

The associations of intermediate to favorable biological CVH profiles with vascular brain health seemed to reverse with advanced age. This is in line with a previous study that showed the age-varying association between hypertension and WMH.^30^ Several reasons might partly contribute to the age-varying associations. First, in the SNAC-K cohort, systolic blood pressure increased with age until ∼80 years and then declined, whereas diastolic blood pressure declined constantly with age.^31^ Similarly, serum total cholesterol has also decreased with age after midlife.^32^ The age variations in these risk factors may lead to differential impacts on brain health from middle age to late life. Furthermore, community-based studies have reported associations of low levels of blood pressure and low-density lipoprotein cholesterol with increased WMH volume in old age.^33-35^ Given that autoregulation of cerebral perfusion is decreased with advanced age, systematic hypoperfusion may be accompanied by cerebral hypoperfusion in very old people,^36^ which may give rise to cerebral ischemic conditions and white matter demyelination.^37^ Moreover, low total cholesterol in late life is associated with impairment of the neuronal myelin structure and synaptic function, which may also accelerate the brain aging process.^38^ Finally, given that high levels of blood pressure, total cholesterol, and fasting glucose are linked with cardiovascular events and increased mortality in advanced age,^39^ the selective survival bias may also weaken the association between biological CVH profiles and vascular brain aging in very old adults.

The genome-wide association studies suggested that the genetic predisposition to higher blood pressure was related to an increased WMH burden.^40^ Similarly, a monozygotic twin-based cohort study (mean age ∼70 years) showed that the shared genetic susceptibility could explain up to 83% of the association between composite cardiovascular risk factor burden (assessed using the Framingham Cardiovascular Risk Score) and WMH load.^41^ These data are in line with our findings that a higher metabolic genetic risk load is related to an increased burden of WMH. Of note, our data further showed that the association between metabolic genetic risk load and WMH burden was present independent of common genetic susceptibility to markers of brain aging (e.g., WMH, PVS, and brain atrophy).

Notably, our study revealed that the association of metabolic genetic predisposition with the progression of WMH was evident among people with unfavorable or intermediate CVH profiles, but not in those with favorable CVH profiles, especially healthy behavior CVH profile. This phenomenon has not been reported before but is in line with the view that healthy behaviors may counteract the detrimental effect of cardiometabolic genetic risk factors and thereby slow down the progression of vascular brain aging.^42,43^ Given the public health relevance, this important finding merits further investigation in large-scale studies of different populations.

The major strength of this study refers to the population-based longitudinal design that integrated comprehensive CVH metric assessments and genetic data of cardiometabolic risk factors with longitudinal structural brain MRI data. However, our study also has limitations. First, we have a relatively small sample. Thus, the statistical power may not be large enough to detect mildly to moderately strong associations. The findings need to be verified in the large-scale cohort studies of different populations. Second, the lack of composite CVH profiles at follow-ups did not allow us to investigate the association of dynamic CVH profiles with brain aging. Third, some imaging markers of vascular brain aging (e.g., cerebral microbleeds and microinfarcts) were not available because of lack of relevant MRI sequences or limited imaging resolution. Fourth, the MRI sample was relatively healthier compared with the whole SNAC-K sample, which might lead to the underestimation of association between composite CVH profiles and vascular brain aging. Finally, the study cohort was derived from a geographic area in central Stockholm where people had relatively high education and high socioeconomic position. This should be kept in mind when generalizing our findings to the genetically and socioeconomically diverse populations.

**Table 3:**
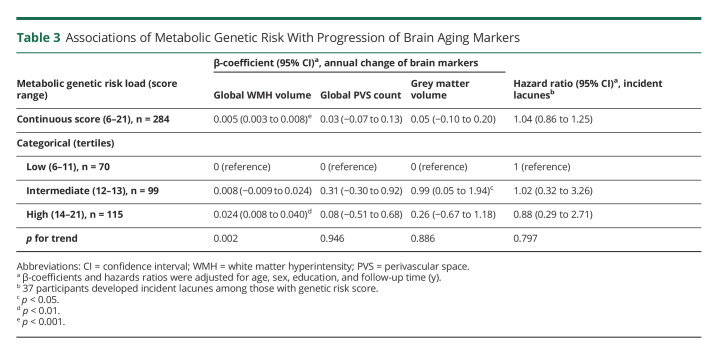
Associations of Metabolic Genetic Risk With Progression of Brain Aging Markers

This population-based cohort study of Swedish older adults indicates that intermediate to favorable global CVH profiles are associated with slower progression of vascular brain aging and that such benefit of intermediate to favorable biological CVH profile is evident only in early old age. In addition, favorable CVH profiles, especially healthy behaviors in CVH profiles, could mitigate accelerated vascular brain aging because of genetic predisposition to metabolic risk factors. These findings highlight the importance of adherence to favorable CVH profiles for brain health in old age. Multidomain intervention studies may help further clarify whether the adherence to favorable CVH profiles, especially health behavior factors, among older adults may help achieve healthy brain aging.

## Glossary

ATC: Anatomical Therapeutic Chemical
GM: gray matter
CVH: cardiovascular health
PVS: perivascular space
SNAC-K: Swedish National Study on Aging and Care in Kungsholmen
SNP: single nucleotide polymorphism
WMH: white matter hyperintensity
